# Exposure to endocrine disruptors promotes biofilm formation and contributes to increased virulence of *Pseudomonas aeruginosa*

**DOI:** 10.1111/1758-2229.13190

**Published:** 2023-08-16

**Authors:** Audrey Thiroux, Jérôme Labanowski, Nicolas Venisse, Stéphanie Crapart, Chloé Boisgrollier, Carlos Linares, Jean‐Marc Berjeaud, Romain Villéger, Alexandre Crépin

**Affiliations:** ^1^ Université de Poitiers, UMR CNRS 7267 Ecologie et Biologie des Interactions Poitiers France; ^2^ Université de Poitiers UMR 7285, Institut de Chimie des Milieux et Matériaux de Poitiers (IC2MP) Poitiers France; ^3^ Université de Poitiers, CHU de Poitiers, INSERM Centre d'investigation clinique CIC1402 Poitiers France

## Abstract

Anthropogenic activities contribute to the spread of chemicals considered as endocrine disruptors (ED) in freshwater ecosystems. While several studies have reported interactions of EDs with organisms in those ecosystems, very few have assessed the effect of these compounds on pathogenic bacteria. Here we have evaluated the impact of five EDs found in aquatic resources on the virulence of human pathogen *P. aeruginosa*. ED concentrations in French aquatic resources of bisphenol A (BPA), dibutyl phthalate (DBP), ethylparaben (EP), methylparaben (MP) and triclosan (TCS) at mean molar concentration were 1.13, 3.58, 0.53, 0.69, and 0.81 nM respectively. No impact on bacterial growth was observed at EDs highest tested concentration. Swimming motility of *P. aeruginosa* decreased to 28.4% when exposed to EP at 100 μM. Swarming motility increased, with MP at 1 nM, 10 and 100 μM (1.5‐fold); conversely, a decrease of 78.5%, with DBP at 100 μM was observed. Furthermore, exposure to 1 nM BPA, DBP and EP increased biofilm formation. *P. aeruginosa* adhesion to lung cells was two‐fold higher upon exposure to 1 nM EP. We demonstrate that ED exposure may simultaneously decrease mobility and increase cell adhesion and biofilm formation, which may promote colonisation and establishment of the pathogen.

## Graphical abstract

This study assessed the effect of EDs (BPA, DBP, EP, MP, and TCS) at environmental concentrations or accumulated in humans on the virulence factors expressed by *P. aeruginosa*. We showed that EDs could modulate the behaviour of *P. aeruginosa* notably BPA, DBP and EP which induce physiological responses like motility, biofilm formation, and ability to adhere to lung cells which could facilitate colonisation of the pathogen in water networks and in humans.

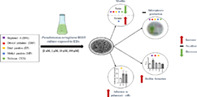

## INTRODUCTION

Anthropogenic activity contributes to the dissemination of chemical substances, some of which have been identified as endocrine disruptors (ED). EDs are chemicals and environmental pollutants defined by the World Health Organization (WHO) as ‘exogenous substances or mixtures that alter the function(s) of the endocrine system and thereby cause adverse health effects in an intact organism or its progeny, or (sub)populations’ (Damstra et al., 2002). Among the main compounds of synthetic origin considered as EDs, bisphenol A (BPA) is used as a plasticiser (polycarbonate production and epoxy resin) in food cans, phthalates are used as plasticisers in the manufacture of polyvinyl chloride (PVC), parabens are used as preservatives in body care products and medicines, and triclosan (TCS) is used in hygiene products for its biocidal properties (Lee et al., 2019). Human activities contribute to the dispersion of these compounds in aquatic environments, encouraging exposure by food/water ingestion, inhalation or skin contact. Adverse effects on reproduction (Ryu et al., 2022), development (Liu et al., 2021) and metabolism (Neier et al., 2020) have been reported. The estrogenic activity exerted by Eds is a major public health problem, correlated with the occurrence of pathologies such as cancers (Calaf et al., 2020) and obesity (Darbre, 2017). Gao et al. demonstrated that bisphenols act as activators of peroxisome proliferator‐activated receptor gamma (PPARγ), at non‐cytotoxic concentrations (1,10,100 μM). Furthermore, exposure to environmentally relevant doses of BPA and bisphenol S (BPS) (50 μg/kg/day) contributes to disruption of lipid metabolism in mice (Gao et al., 2020). Hu et al. demonstrated that parabens activated PPARγ in the adipocyte signalling pathway, and their potency increased as the length of the linear alkyl chain increased (butylparaben) (Hu et al., 2013).

Use of these synthetic substances is regulated by Registration, Evaluation, Authorization and restriction of CHemicals in Europe. Use of BPA is banned in products for children (−3 years) in the European Union (2018). Its use in food containers is banned in France but still under discussion at the EU level. In 2023, EFSA re‐evaluated tolerable daily intake of BPA in food containers from 4 mg/kg/day to 0.2 ng/kg/day (EFSA, 2023). Use of phthalates such as di(2‐ethylhexyl) phthalate (DEHP), dibutyl phthalate (DBP), benzyl butyl phthalate (BBP), diisobutyl phthalate (DIBP), must be less than 0.1% in electrical and electronic equipment, toys and childcare articles. They are subject to authorization in medical devices. In France, DEHP is prohibited in tubing of medical devices in paediatrics. Parabens are regulated at a maximum concentration of 0.4% alone and 0.8% in combination with cosmetic products. Triclosan is limited to a maximum concentration of 0.3% in personal care products, but banned in food containers. Although measures have been taken to limit their use, EDs persist in the environment, particularly in aquatic resources. In Europe, Jonkers et al., identified EDs in Portuguese freshwater, such as MP and BPA, with respective mean concentrations of 8.8 ng/L, and 212 ng/L (Jonkers et al., 2010). In Asia, Zhang et al, detected bisphenols in Chinese source water, at the following mean concentrations: BPA (12.8 ng/L), bisphenol AF (BPAF) (3.0 ng/L), bisphenol B (BPB) (1.0 ng/L), bisphenol E (BPE) (0.98 ng/L), bisphenol F (BPF) (2.18 ng/L), and BPS (1.1 ng/L) (Zhang et al., 2019). In Canada, maximum concentration of BPA was 6370 ng/L with a mean value of 101 ng/L in surface water (Gewurtz et al., 2021). Aquatic resources not only contain EDs but are also the ecological niche for numerous microorganisms, including the pathogenic bacterium *Pseudomonas aeruginosa*.

*P. aeruginosa* is an opportunistic gram‐negative bacillus, ubiquitous in the environment and often found in freshwater resources, either under free‐living (planktonic) or biofilm (sessile) form (Li et al., 2017). The biofilm formed by the bacteria is a community of microorganisms embedded in a matrix of exopolysaccharides, which adheres to any surface, living or non‐living. *P. aeruginosa* matrix includes not only exopolymers coded by *Pel*, *Psl*, and *Alg* operons but also eDNA, lipids and proteins that help to maintain and structure biofilm. Biofilms found in aquatic environments can constitute reservoirs of pathogen agents (Wingender & Flemming, 2011). In addition, chronic exposure to chemical pollutants contributes to selective pressure for antibiotic resistance in biofilm (Flores‐Vargas et al., 2021). Furthermore, biofilm formation is involved in corrosion (Ogawa et al., 2020) and promotes bioaccumulation of chemical compounds (Huerta et al., 2016). Biofilm formation of *P. aeruginosa* plays a significant role in transmission and persistence of the bacteria in humans. Persistent infections caused by biofilm‐growing *P. aeruginosa* strains, such as catheter or lung infections, are mostly due to biofilm tolerance towards antimicrobial agents (Billings et al., 2013) and the immune system (Farrant et al., 2020). It is responsible for a wide range of acute and chronic infections, especially lung infections in immunocompromised patients (Liao et al., 2022), such as cystic fibrosis, for which it is the main morbidity agent (Pang et al., 2019). Water is a major contributor to the spread and transmission of the pathogen in the hospital environment, including many inanimate surfaces such as medical devices or water distribution systems (plumbing). In order to limit the establishment of biofilm and the risks of infection, the use of anti‐biofilm natural agents such as baicalin, which inhibit Quorum Sensing detection (Luo et al., 2017), antimicrobial/adhesive surfaces such as polyethylene glycol‐linked polypeptide antimicrobial (Gao et al., 2017), biomaterialials such as a natural polymer (dextran, algal polysaccharide) and methods of plumbing water disinfection such as chlorination, copper–silver ionisation, ozonation, UV, temperature control are used. Moreover, photodynamic treatment on mature biofilms in patients would be a safe alternative because it is durable, non‐toxic and minimally invasive in cases of chronic infection (Elkihel et al., 2021; Warrier et al., 2021). Among the other virulence factors expressed by *P. aeruginosa* and involved in infection processes, pyocyanin is a secondary metabolite toxic for eukaryotic cells that suppresses the host immune response while contributing to increased IL‐8 production (Chai et al., 2013). On the other hand, pyocyanin is able to induce apoptosis of epithelial cells by cytotoxic formation of free radicals. Another metabolite produced by the pathogen is pyoverdine, a preponderant virulence factor in acute infections. It is a siderophore that ensures the acquisition of iron and is essential for the growth of the bacteria (Durán et al., 2022). In addition, the presence of a single polar flagellum allows the bacteria to move and colonise an ecological niche. It is highly immunogenic and provides a survival advantage in chronic infections (Balloy et al., 2007; Valentin et al., 2022). Many of these intrinsic factors can be regulated by QS, a chemical communication process that *P. aeruginosa* uses to control collective behaviours including virulence and biofilm formation (Sousa & Pereira, 2014). Furthermore, hormones produced by humans are able to modulate *Pseudomonas* phenotypes by influencing QS, promoting virulence factor expression. For example, serotonin acts as a signalling molecule via the detection of QS by stimulating the production of virulence factors in an infected mouse model (Ahmed et al., 2019; Knecht et al., 2016). Epinephrine is able to modulate motility, increase adhesion and biofilm formation of *P. aeruginosa* H103 at low concentrations (1 and 10 μM) (Cambronel et al., 2019). These examples highlight the existence of inter‐kingdom signalling (Yong et al., 2018). Phenotypic modulations of *P. aeruginosa* by human hormones have raised many questions regarding the interactions between the bacteria and EDs because they are analogous to human hormones. Given the ability of certain human hormones found in water environments to modulate the virulence of *P. aeruginosa*, it seems required to evaluate the impact of EDs on the virulence of this pathogen.

Limited information is available in the literature regarding the consequences of ED exposure on the virulence of pathogens. It has been shown that TCS promotes the colonisation by *Staphylococcus aureus* of the nasal cavity (Syed et al., 2014). A study conducted on *Streptococcus mutans*, the causative agent of dental caries, showed that exposure to sub‐inhibitory concentrations of TCS increases biofilm formation (Bedran et al., 2014). Moreover, we recently demonstrated that phthalates and their substitutes can affect *P. aeruginosa* physiology. In particular, we have demonstrated that exposure to nanomolar, micromolar and millimolar concentrations of several phthalates could increase *P. aeruginosa* biofilm formation (Louis et al., 2022).

To date, the effect of EDs found in water on the virulence of this bacteria has been poorly studied. The innovative nature of this work consists in the evaluation of several families of EDs (plasticizers, preservatives, biocides) and their effects on the physiology and behaviour of the human pathogen, *P. aeruginosa*, whereas other studies only study one family. Here we have evaluated the impact of five EDs exposure, bisphenol A (BPA), dibutyl phthalate (DBP), ethylparaben (EP), methylparaben (MP) and triclosan (TCS) on several virulence factors of the pathogen *P. aeruginosa*. Our analysis focused on ability to form biofilm, siderophore production, motility and ability to adhere and infect human lung epithelial cells. In addition, this work indicates the ED concentrations found in French aquatic resources. Thereby, this work is part of a preventive approach aimed at informing the scientific community on spread of EDs in the environment and on the risks and consequences of chronic exposure on the virulence of pathogens.

## EXPERIMENTAL PROCEDURES

### Endocrine disruptors

Bisphenol A, dibutyl phthalate, ethylparaben (ethyl 4‐hydroxybenzoate), methylparaben (methyl 4‐hydroxybenzoate) and triclosan were purchased from Sigma‐Aldrich (St. Louis, MI, USA). The concentrations used in the experiments were 100, 10, 1 μM, and 1 nM for each ED. EDs have been prepared and solubilised in dimethylsulfoxyde (DMSO, ≥ 99.9%, Thermo Fisher Scientific).

### Bacterial strain and culture conditions

The bacterium used in this study was *P. aeruginosa* H103, a prototroph of the PAO1 wild‐type strain. Bacteria were precultured for 24 h at 37°C under agitation (180 rpm) in Lysogeny Broth (LB) medium with 10 g/L Tryptone, 5 g/L yeast extract, and 10 g/L NaCl.

### Cell culture

The A549 lung epithelial cell line was obtained from the American Type Culture Collection (ATCC CCL‐185). Cells were cultured in 75 cm^2^ flasks (Thermo Fisher Scientific) in Dulbecco's Modified Eagle Medium supplemented with 10% Fetal Bovine Serum and 1% penicillin/streptomycin (P/S) at 37°C with 5% CO_2_ (all from Gibco™, Grand Island, NY, USA). A549 infection assay (adhesion, invasion and intracellular multiplication) was assessed in 24‐well plates.

### Determination of EDs in the aquatic resources of Nouvelle‐Aquitaine region (France)

The measurements were performed in the Nouvelle‐Aquitaine region over the period 2015–2020. The data provided come from the databases of the French water agency ‘Agences de l'Eau’. Concentrations were measured in rivers, streams, dams, ponds, canals and lakes. The sampling sites as well as average ED concentrations values are reported in Table S1.

### Virulence factors

#### Growth kinetics

A culture of *P. aeruginosa* H103 was grown overnight in LB medium at 37°C with shaking at 180 rpm. The bacterial suspension was adjusted to an A_600nm_ value of 0.05 and 200 μL was added into a 96‐well plate in presence of EDs at concentrations of 1 nM, 1, 10, and 100 μM with DMSO as a control. Growth was monitored and measured for 24 h at 37°C with shaking (180 rpm) to A_600nm_ using the TECAN Infinite® 200 PRO plate reader (Tecan Group Ltd.) controlled by i‐control™ software. Each experiment was performed three times with at least three biological replicates per test.

#### Motility tests

Swim motility was performed in LB agar medium with 0.3% agar (Fisher Scientific) supplemented with the following concentrations of EDs: 1 nM, 1, 10, and 100 μM. A colony of *P. aeruginosa* was then picked with a sterile tip and inoculated onto the dish by pricking the surface of the medium. The swim plates were placed at 37°C for 18 h.

Swarm motility, LB agar medium with 0.6% agar was supplemented with ED concentrations as described previously. From an overnight culture adjusted to A_600nm_ value of 0.05, then 5 μL of culture was placed in the centre of the dish. The plates were incubated at 37°C for 18 h. The experiments were performed in triplicate and repeated three times independently. Results were normalised to the (CtrL) and expressed as a percentage of measured area (% cm^2^) using ImageJ software.

#### Siderophore production

Pyocyanin production was quantified from *P. aeruginosa* culture after 24 h of exposure to EDs (BPA, DBP, EP, MP and TCS) at 1 nM, 1, 1, and 100 μM concentrations as described previously (Louis et al., 2022). Cultures were performed in King A medium (20 g/L peptone protease, 10 g/L glycerol, 10 g/L K_2_SO_4_, 1.4 g/L MgCl_2_) and adjusted to pH 7.2. Cultures were centrifuged at 5000 × *g*/10 min/RT. 2 mL of supernatant was added to 2 mL of chloroform. Pyocyanin from the chloroform phase was then reextracted into 1 mL of 0.5 M HCl giving a pink to deep red solution. Thereafter, pyocyanin production was measured by spectrophotometry at 520 nm and normalised to the bacterial growth measured at 600 nm. The experiment was performed three times independently and the results were reported as a percentage of pyocyanin production compared to control (DMSO).

Pyoverdine production was assessed as described previously (Louis et al., 2022) in King B medium (20 g/L peptone protease, 10 g/L glycerol, 1.5 g/L K_2_HPO_4_, 1.5 g/L MgSO_4_) and adjusted to pH 7.2 in the same conditions as described for pyocyanin production. After 24 h, cultures were centrifuged at 5000 × *g*/10 min/RT. Pyoverdine production was measured at 400 nm and normalised to the bacterial growth (measured at 600 nm). The experiment was performed three times independently and the results were reported as a percentage of pyoverdine production compared to control (DMSO).

#### Biofilm formation

The effect of EDs on biofilm formation was tested for each ED concentrations and according to three exposure modes: exposure of planktonic cells during preculture; continuous exposure of both planktonic cells during preculture and during biofilm formation; and exposure only during biofilm formation. First, precultures and biofilm formation assay of *P. aeruginosa* were performed in absence or presence of EDs at 1 nM, 1, 10, and 100 μM. The precultures were performed in a final volume of 10 mL adjusted to an A_600nm_ value of 0.05 and incubated at 37°C for 24 h. Bacteria were pelleted at 5000 × *g*/10 min/RT. The supernatants were removed and the pellets were resuspended in 10 mL of LB medium. Bacterial suspensions were adjusted to an A_600nm_ value of 0.05 with or without addition of EDs. The bacterial suspensions were then placed in 96‐well plates at a final volume of 200 μL. After 2 h of incubation at 37°C without shaking, the wells were washed with PBS to remove planktonic bacteria and to keep only the bacteria adhered to the bottom of the plate. Depending on the exposure pattern, LB medium supplemented or not with EDs (at different concentrations) was added to the wells. The plate was then placed at 37°C for 24 h. After removing supernatants, biofilm formation was quantified by adding 200 μL of 0.3% crystal violet to each well and incubation of the plate for 15 min under orbital shaking/300 rpm/RT. Wells were washed three times with PBS to remove excess CV. Then 200 μL of 96% ethanol (Thermo Fisher Scientific, Germany) was added to each well in order to dissolve the dye and incubated for 15 min under orbital shaking/300 rpm/RT. Finally, biofilm formation was measured by absorbance measurement at 595 nm using TECAN Infinite® 200 PRO plate reader.

#### Adhesion and invasion assay

The effect of EDs on bacterial invasion was determined using the gentamicin protection assay as described before with some modifications (Chi et al., 1991). A549 cells were plated in 24‐well plates at 1.10^5^ cells/well and incubated for 24 h at 37°C/5% CO_2_. In parallel, a culture of *P. aeruginosa* adjusted to an A_600nm_ value of 0.05 was exposed for 24 h/37°C/180 rpm to EDs; BPA, DBP, and EP at concentrations of 1 nM and 100 μM. A549 cells were washed twice with PBS (Gibco™, Paisley, Scotland, UK) to remove all traces of antibiotics (P/S). The cultures of *P. aeruginosa* exposed to EDs were centrifuged and then transferred into DMEM medium (Gibco™, Grand Island, NY, USA) 10% SVF without antibiotic and maintained exposure to the tested EDs. A549 cells were infected with the cultures of *P. aeruginosa* exposed to EDs at a multiplicity of infection of 10. The plates were centrifuged at 36 × *g* for 10 min at RT and then incubated at 37°C/5% CO_2_. For the adhesion assay, after 3 h of infection, cells were washed twice with PBS and lysed with 0.1% Triton X‐100 (Sigma‐Aldrich). The suspension was serially diluted and cultured on LB agar plates for viable counting of bacteria after overnight incubation at 37°C. The colony‐forming units (CFU) were counted to establish the number of adhered bacteria (*N* (Total Adhesion) – *N* (Invasion)). Each condition was then normalised by comparison with the control mean and expressed as a percentage of adhesion.

For the invasion assay, after 3 h of infection, gentamicin (200 μg/mL) was added for 1 h to kill the extracellular bacteria (T4h Post infection). As described previously for the adhesion assay, the same procedure was followed for the lysis and counting bacteria. The CFUs counted are reported as internalized bacteria. For the intracellular multiplication assay, as in the invasion assay after 3 h of infection, a one‐hour treatment with gentamicin (200 μg/mL) was added and maintained during the 21 h incubation (T24h Post Infection). CFU counted are reported as “persistent” bacteria in the intracellular compartment. Each condition was normalized by comparison with the control mean and expressed as percentage invasion/multiplication.

#### Quantification of virulence gene expression by RT‐qPCR

Virulence gene expression was quantified by RT‐qPCR from bacterial cultures exposed to EDs. Bacteria were harvested by centrifugation at 5000 × *g*/5 min/4°C after 2, 5, and 14 h of culture in presence of EDs at 1 nM, 1, 10, and 100 μM. Bacterial pellets were then stored and placed at −80°C for further RNA extraction. RNA was extracted using the RNeasy Mini kit (QIAGEN, Germantown, MD, USA) according to manufacturer's recommendations. DNAse treatment was then performed using the Turbo DNA Free Kit (ThermoFisher Scientific, Carlsbad, CA, USA), to remove residual gDNA. RNA purity and concentration were measured using NanoDrop™ (ThermoFisher Scientific, Wilmington, DE USA) and normalized for the reverse transcription step. RNAs were reverse transcribed using GoScript™ Reverse Transcriptase according to manufacturer's recommendations using random primers (PROMEGA, Madison, WI, USA). qPCR was performed on a LightCycler® 480 thermal cycler (Roche, Rotkreuz, Switzerland) using the Takyon™ No ROX SYBR 2X MasterMix blue dTTP kit (Eurogentec, Seraing, Belgium) according to the supplier's recommendations. The primers used are presented in Table S2. The reaction mix contained 8 μL of Takyon mix 1X including 0.3 μM of forward/reverse primers and nuclease‐free water then 2 μL of cDNA. The qPCR cycling programe was as follows: 95°C for 5 min followed by 45 cycles at 95°C for 10 s, 58°C for 10 s, 72°C for 10 s, and a dissociation cycle at 95°C for 5 s, followed by 65°C for 1 min. The level of gene expression was calculated based on the threshold cycle (CT) normalised to the CT of the reference gene *rpoS*. The relative quantification of the virulence genes was compared to the values of the control ΔCT (DMSO). The relative expression data were calculated by ^2−ΔΔ^CT method and the experiment were performed three times independently for each sample. Forward and reverse primers for each virulence gene of *P. aeruginosa* are presented in Table S2.

### Statistics

Statistical analysis was performed using GraphPad Prism (GraphPad Prism 8.0.1; GraphPad Software, San Diego, CA, USA). All data represent at least three independent experiments and are expressed as means ± deviations from the mean (SEM) unless otherwise stated. An ANOVA test, combined with an uncorrected Fisher's test was performed on the biofilm formation and relative virulence gene expression experiments. For the other experiments, the Kruskal–Wallis test was used and combined with an uncorrected Dunn's test. Unless otherwise stated, *p*‐values are indicated in all figures and tables as follows: *, *p <* 0.05; **, *p <* 0.01; and ***, *p <* 0.001.

## RESULTS

### Contamination of aquatic resources with low concentrations of endocrine disruptors

**FIGURE 1:**
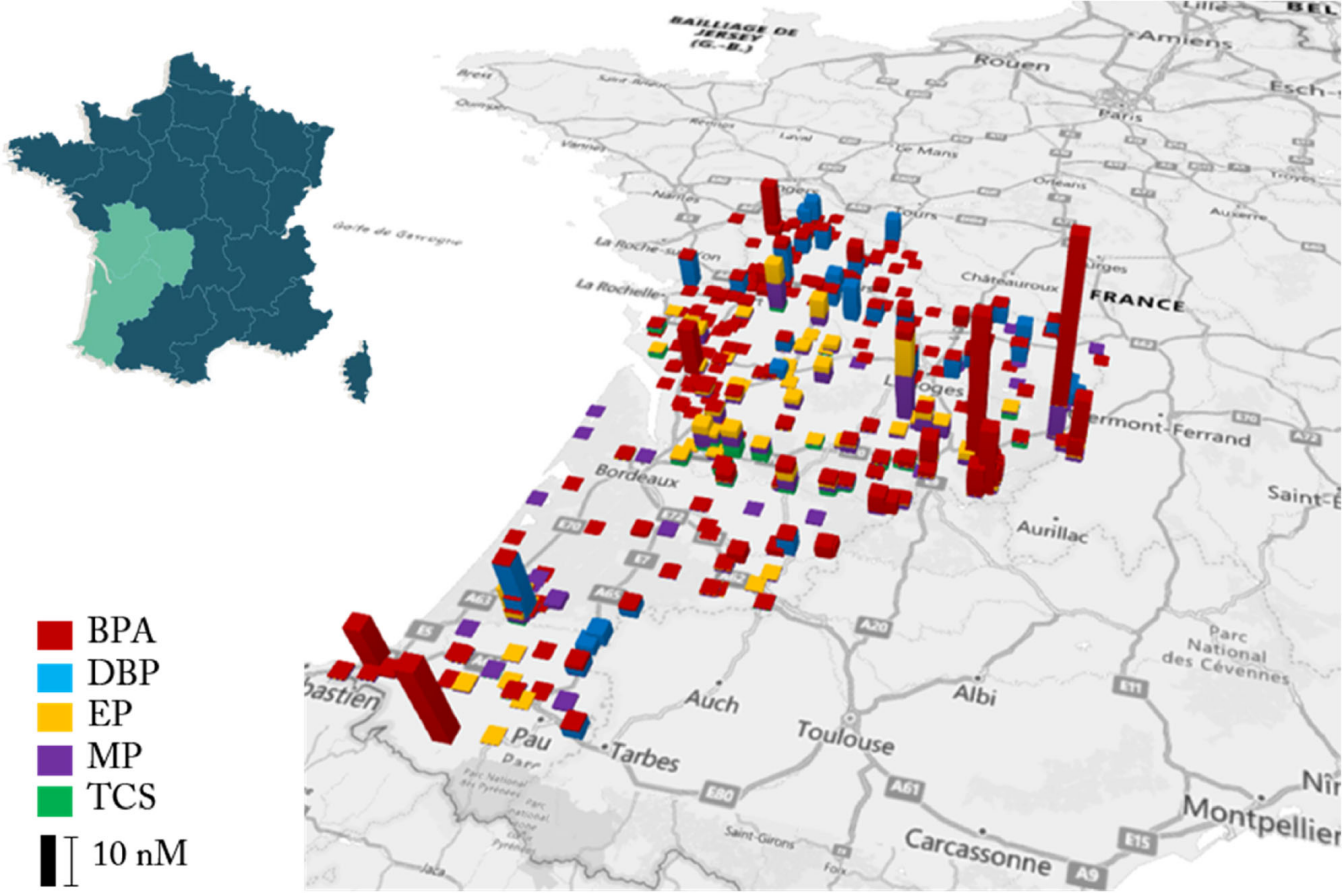
Map of endocrine disruptors found in the aquatic environment of the Nouvelle‐Aquitaine region between 2015 and 2020. The data are from the French Water Agency. The measurements are expressed in nM. The colours of the bars represent the different endocrine disruptors; red, BPA, blue, DBP, yellow, EP, purple, MP and green, TCS identified and measured.

In the Nouvelle‐Aquitaine region (France), numerous water samples were collected between 2015 and 2020 from rivers, streams, dams, ponds, canals and lakes and the concentrations of chemical pollutants were determined by LC–MS by the French Water Agencies (Agences de l'Eau). We analysed the data regarding BPA, DBP, EP, MP, and TCS and found a total of 21,181 measurements that detected at least one of these compounds. Among these, 6563 measurements for BPA were above the limit of quantification (LOQ) with a detection frequency of 13.29%, 4529 measurements for DBP were above the LOQ with a frequency of 1.15%, 3398 measurements for EP were above the LOQ with a frequency of 4.47%, 4529 measurements for MP were above the LOQ with a frequency of 10.93% finally 3498 measurements for TCS were above the LOQ with a frequency of 1.09%. The measured concentrations for each ED are presented in Figure 1. These data showed that BPA and DBP are more frequently found in aquatic resources than EP, MP and TCS. The minimum, maximum and average concentration values determined for each of the ED are reported in Table 1. The concentrations of BPA, DBP, EP, MP, and TCS found in the aquatic resources of the Nouvelle‐Aquitaine region are in the nanomolar range. The average concentrations measured were significantly higher for BPA and DBP than for EP, MP and TCS. Thus, BPA was measured between 0.06 and 31.10 nM with an average of 1.13 nM, DBP between 1.43 and 10.58 nM with an average of 3.58 nM, EP between 0.06 and 7.22 nM with an average of 0.53 nM, MP between 0.06 and 9.20 nM with an average of 0.69 nM and TCS between 0.03 and 2.72 nM with an average of 0.81 nM. The concentrations obtained in the aquatic resources of the Nouvelle‐Aquitaine region provide evidence of permanent contamination of the water with EDs.

**TABLE 1:** Molar concentration of endocrine disruptors in aquatic resources in the Nouvelle‐Aquitaine region between 2015 and 2020.

Endocrine disruptors	Mean molar concentration (nM)	Minimum values (nM)	Maximum values (nM)
BPA	1.13	0.06	31.10
DBP	3.58	1.43	10.58
EP	0.53	0.06	7.22
MP	0.69	0.06	9.20
TCS	0.81	0.03	2.72

*Note*: The concentrations were averaged and converted into molar concentrations using data from the Water Agency.

### *EDs do not affect the growth of* P. aeruginosa

**FIGURE 2:**
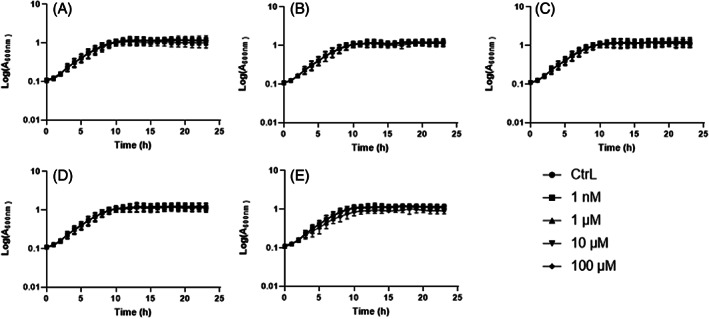
Growth kinetics of *Pseudomonas aeruginosa* exposed to endocrine disruptors. *P. aeruginosa* was cultured in LB medium in presence of 1 nM, 1, 10, and 100 μM of BPA (A), DBP (B), EP (C), MP (D) and TCS (E) or DMSO as control (CtrL). Optical density measurements at 600 nm were performed every hour with 10 s of shaking at 432 rpm during 24 h at 37°C in 96‐well plates. Results are presented as mean ± SEM of log (A_600nm_) from three independent experiments.

The growth of *P. aeruginosa* was assessed in LB medium in the presence of various concentrations of BPA, DBP, EP, MP, TCS or the solvent (DMSO) as the control. The growth of *P. aeruginosa* was monitored by measuring the optical density at 600 nm for 24 h in 96‐well microplates. The growth curves are presented in Figure 2. Our results indicated that none of the EDs, whatever the concentration, affected the growth of *P. aeruginosa* compared to the control. However, 100 μM TCS slightly slows down the growth of the bacteria.

### *Modulation of* P. aeruginosa *swim and swarm motility when exposed to ED*

**FIGURE 3:**
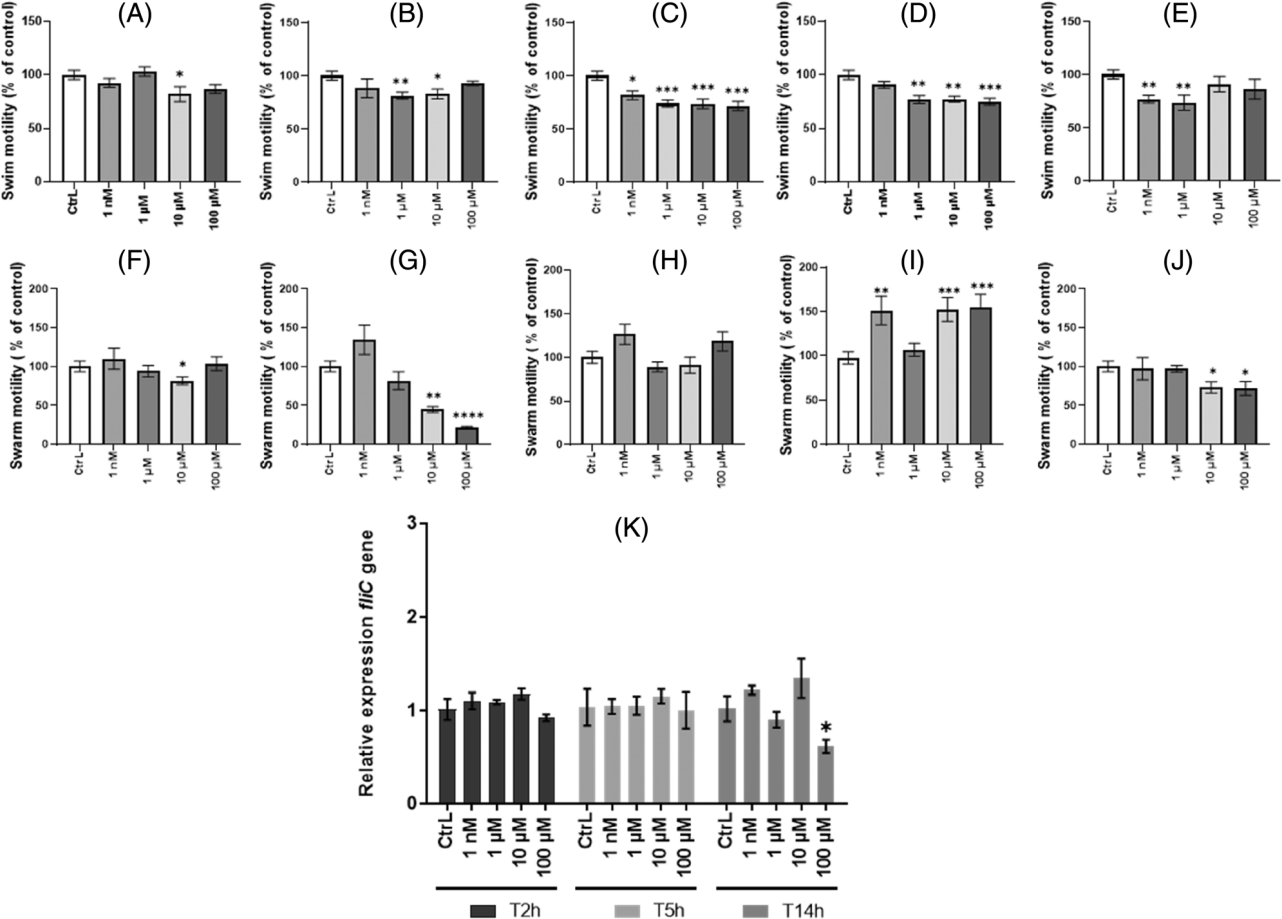
Evaluation of *Pseudomonas aeruginosa* motility in presence of endocrine disruptors. (A)–(E) Swimming motility was assessed in LB containing 0.3% agar in the presence of BPA (A), DBP (B), EP (C), MP, (D) and TCS (E) at concentrations of 1 nM, 1, 10, and 100 μM or DMSO as control (CtrL). (F)–(J) Swarming motility was assessed in LB containing 0.6% agar in the presence of BPA (F), DBP (G), EP (H), MP (I) and TCS (J) at concentrations of 1 nM, 1, 10, and 100 μM or DMSO as control (CtrL). Results are normalised to the (CtrL) and expressed as percentage of measured area (% cm2) using ImageJ software. Results correspond to as mean ± SEM of three independent experiments. Statistical analysis was performed using nonparametric Kruskal–Wallis test, * *p <* 0.05; ** *p <* 0.01; *** *p <* 0.001. Relative expression of virulence gene (K) *fliC* in *P. aeruginosa* after exposure to TCS was determined by RT‐qPCR. *P. aeruginosa* was cultured in LB medium at 37°C under agitation in presence or not of 1 nM, 1, 10, and 100 μM of EDs. Bacterial pellets were harvested at *T* = 2 h, *T* = 5 h, and *T* = 14 h mimicking the different growth phases. Results are presented as mean ± SEM of triplicate. Statistical analysis was performed using 2Way ANOVA, * *p <* 0.05; ** *p <* 0.01; *** *p <* 0,001, **** *p <* 0.0001.

We next evaluated *P. aeruginosa* swim and swarm motilities on agar plates when exposed to EDs. The diameter displacement areas of *P. aeruginosa* on agar plates, containing ED at the tested concentrations, were measured and compared to the control obtained with DMSO only. Overall, we observed a decrease of swimming of the bacteria when exposed to EDs (Figure 3). However, this decrease depended on the ED concentration tested. A significant decrease of 17.8% in swimming was observed only with 10 μM BPA (Figure 3A). Similarly, significant decreases of 18.4% and 16.9% in swimming were observed with 1 and 10 μM DBP, respectively (Figure 3B). For all the concentrations of EP tested, a decrease in a dose‐dependent manner was observed for 1 nM, 1, 10, and 100 μM, respectively (Figure 3C), with reductions of 18.3%, 25.9%, 26.3%, and 28.4%. Swimming at 1, 10, and 100 μM MP concentrations were significantly reduced by 22.7%, 22.4%, and 24.8% respectively (Figure 3D). Finally, swimming at 1 nM and 1 μM TCS was reduced by 22.8% and 26.2%, respectively (Figure 3E). The swimming areas of *P. aeruginosa* appeared circular for BPA, DBP, EP, and MP as observed in the control. Surprisingly, a dendritic‐like area was observed in the presence of 100 μM TCS, which is usually found for swarm motility.

The swarming evaluation, which was assessed on 0.6% agar medium, showed that the surface displacement of *P. aeruginosa* was modulated depending on the ED tested. Swarm motility appeared significantly decreased by 18.4% when exposed to 10 μM BPA (Figure 3F) and by 55% and 78.5% when exposed to 10 and 100 μM DBP, respectively (Figure 3G). Similarly, TCS at 10 and 100 μM significantly reduced by 26.7% and 28.0% the swarming motility of the bacteria (Figure 3J). In contrast, swarming was significantly increased in the presence of 1 nM, 10 and 100 μM of MP (Figure 3I) by 51.2%, 52.3%, and 54.1%, respectively, while no modification of swarming with EP was observed (Figure 3H). The *P. aeruginosa* swarming areas appeared dendritic‐like for all the EDs tested and whatever the concentration, except for 100 μM DBP for which the area appeared circular (data not shown).

Taken together, the assays indicate an overall decrease in swim and swarm motility of *P. aeruginosa* exposed to EDs. Since motility involves appendages such as the flagellum, we quantified the expression of *fliC* by RT‐qPCR, a gene involved in flagellum production, when the bacteria were exposed to EDs in planktonic cultures. Bacterial cultures exposed to EDs were harvested at 2, 5, and 14 h corresponding to the lag phase, exponential phase and stationary phase respectively of the bacteria growth curve (Figure 2). Exposure to 100 μM TCS significantly decreased expression (−14%) of the *fliC* after 14 h of culture (Figure 3K). Nevertheless, the expression of *fliC* in the presence of BPA, DBP, EP, and MP was not affected (data not shown). Taken together, these results could indicate a decrease in the virulence of the bacteria.

### Pyocyanin and pyoverdine production is not modulated by EDs

**FIGURE 4:**
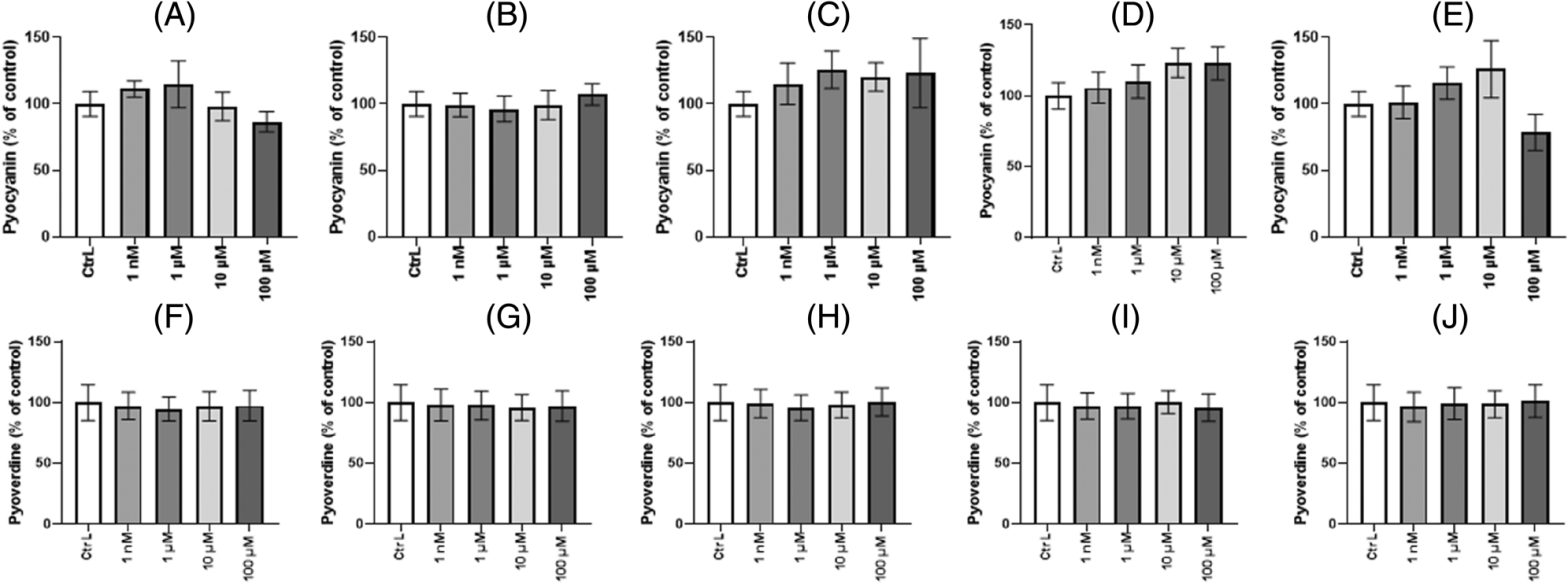
Pyocyanin and Pyoverdine production by *Pseudomonas aeruginosa* exposed to endocrine disruptors. Pyocyanin was assessed in liquid medium King A and exposed to EDs, (A) BPA, (B) DBP, (C) EP, (D) MP and (E) TCS for 24 h. The A520/A600 ratio was measured for all conditions tested and expressed as a percentage of control. Results are presented as mean ± SEM of three independents replicat. Statistical analysis was performed using the nonparametric Kruskal–Wallis test. Pyoverdine was assessed in liquid medium King B and exposed to EDs, (F) BPA, (G) DBP, (H) EP, (I) MP, and (J) TCS for 24 h A400/A600 ratio was measured for all conditions tested and expressed as a percentage of control. Results are presented as mean ± SEM of three independents replicat. Statistical analysis was performed using the nonparametric Kruskal–Wallis test.

The production of pyocyanin and pyoverdine after 24 h exposure to EDs was evaluated using King A and King B media, respectively, with DMSO as control. No significant change in expression was observed in the production of pyocyanin and pyoverdine (Figure 4). Nevertheless, pyocyanin production tended to decrease (−22%) in the presence of 100 μM TCS (Figure 4E) causing a loss of pigmentation of the extracellular medium. This observation suggests that the biocidal activity of TCS at high concentrations (100 μM) disrupts production of the pyocyanin pigment.

### BPA, DBP, and EP enhanced biofilm formation at environmental concentrations

**FIGURE 5:**
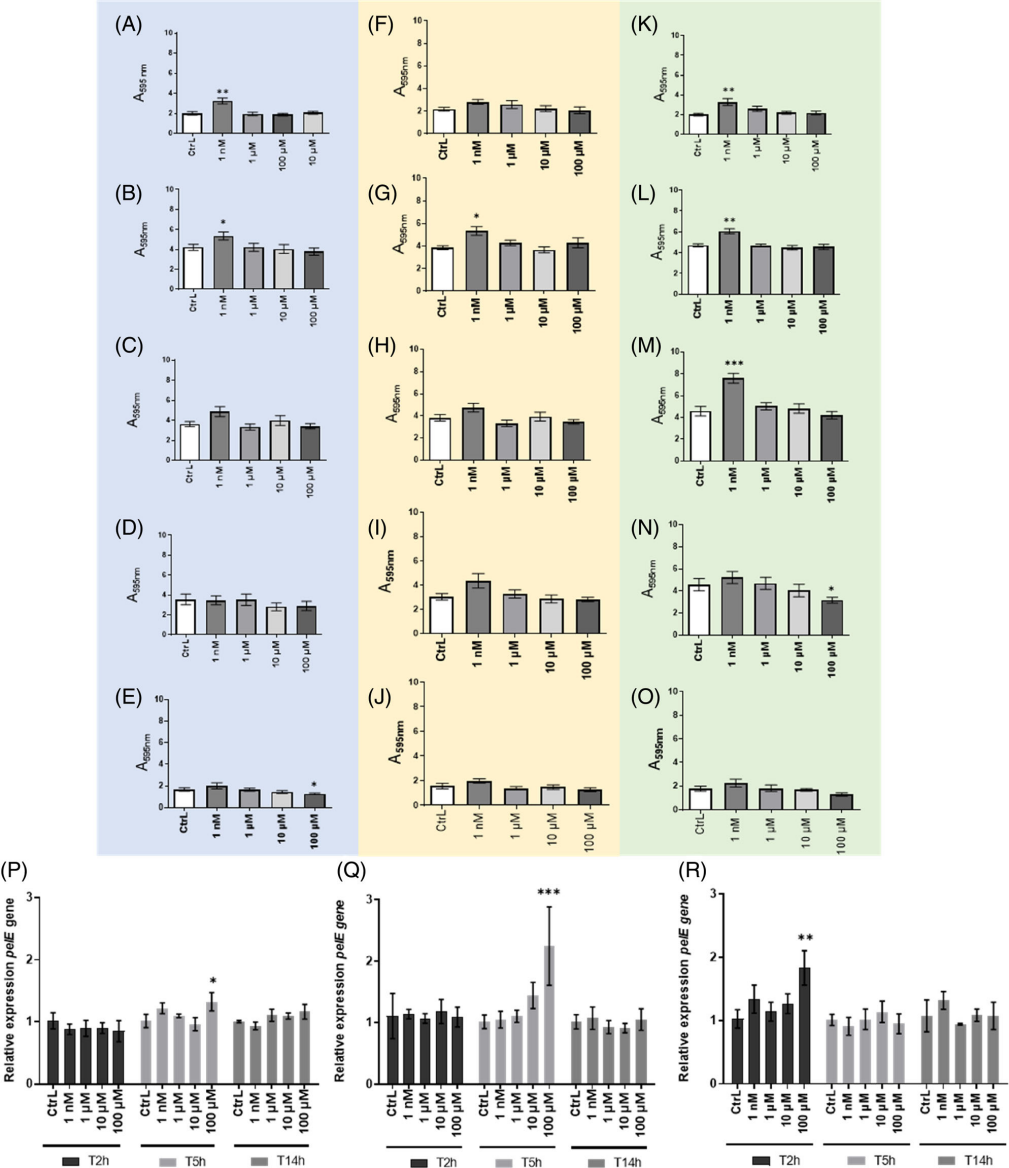
Effect of endocrine disruptors on the biofilm formation of *P. aeruginosa*. (A‐O) Biofilm formation was assessed in the presence of EDs according to three modes of exposure: exposure to EDs only planktonic culture (blue background), recovered and adjusted to an A_600nm_ value of 0.05 and placed in plates BPA (A), DBP (B), EP (C), MP (D), TCS (E). Continuous exposure to EDs during planktonic culture and during biofilm formation (orange background) BPA (F), DBP (G), EP (H), MP (I), TCS (J). Exposure to EDs only at the time of biofilm formation (green background) BPA (K), DBP (L), EP (M), MP (N), TCS (O). Biofilms formed were quantified by CV staining and results are presented as mean ± SEM of triplicate. Statistical analysis was performed using nonparametric Kruskal–Wallis test, * *p <* 0.05; ** *p* < 0.01; *** *p* < 0.001. (P)–(R) Relative expression of virulence gene *pelE* of *P. aeruginosa* after exposure to (P) BPA, (Q) EP or (R) TCS. *P. aeruginosa* was cultured in LB medium at 37°C under agitation in presence or not of 1 nM, 1, 10, and 100 μM of EDs. Bacterial pellets were harvested at *T* = 2 h, *T* = 5 h, and *T* = 14 h mimicking the growth phases. Results are presented as mean ± SEM of triplicate. Statistical analysis was performed using 2Way ANOVA, * *p <* 0.05; ** *p <* 0.01; *** *p <* 0.001, **** *p <* 0.0001.

To assess the effect of EDs on the ability of *P. aeruginosa* to form a biofilm, the bacteria were exposed to EDs according to three modes of exposure (see ‘Experimental Procedures’ section) and cultured for 24 h in 96‐well microplates in LB medium, supplemented or not with EDs (Figure 5). When preculture only was exposed to EDs (Figure 5A–E, light blue background), a significant increase in biofilm formation was observed in the presence of BPA (Figure 5A) and DBP (Figure 5B) only at the lowest concentration (1 nM) with respectively 1.62 and 1.27‐fold increase. Conversely, a significant decrease in biofilm formation was observed in the presence of 100 μM TCS (Figure 5E). No significant effect was observed in the presence of EP (Figure 5C) and MP (Figure 5D) regardless of the concentration tested. The continuous exposure experiments (Figure 5F–J, light orange background) revealed a significant increase in biofilm formation for DBP only (Figure 5G) at 1 nM (1.38‐fold). No significant difference was observed for biofilm formation in the presence of BPA (Figure 5F), EP (Figure 5F), MP (Figure 5I), and TCS (Figure 5J) whatever the concentration. Finally, the exposure to EDs only during biofilm formation (Figure 5K–O, light green background), induced a 1.64‐fold increase in biofilm formation when exposed to 1 nM BPA (Figure 5K), with a 1.29‐fold increase with 1 nM DBP (Figure 5L) and 1.66‐fold with 1 nM EP (Figure 5M). However, a significant decrease with 100 μM MP was observed (Figure 5N). Finally, no significant effect of TCS on biofilm formation was observed (Figure 5O). Taken together, these results suggest that BPA, DBP and EP could increase biofilm formation at 1 nM, which refers to the concentration found in environmental water. In parallel, evaluation of the expression of the *pelE* gene coding a sucrose synthase involved in biofilm formation was performed (Figure 5O, P). The presence of BPA (Figure 5P), EP (Figure 5Q) and TCS (Figure 5R) in planktonic culture showed increased expression of the *pelE* gene, which varied according to harvest time (2, 5, and 14 h). Thus, the expression level of the *pelE* was significantly increased in a 2 h culture with 100 μM TCS (1.79 fold) and in a 5 h culture with 100 μM BPA (1.31 fold) and 100 μM EP (2.22 fold).

### P. aeruginosa *adhesion to lung cells increases in the presence of EDs*

**FIGURE 6:**
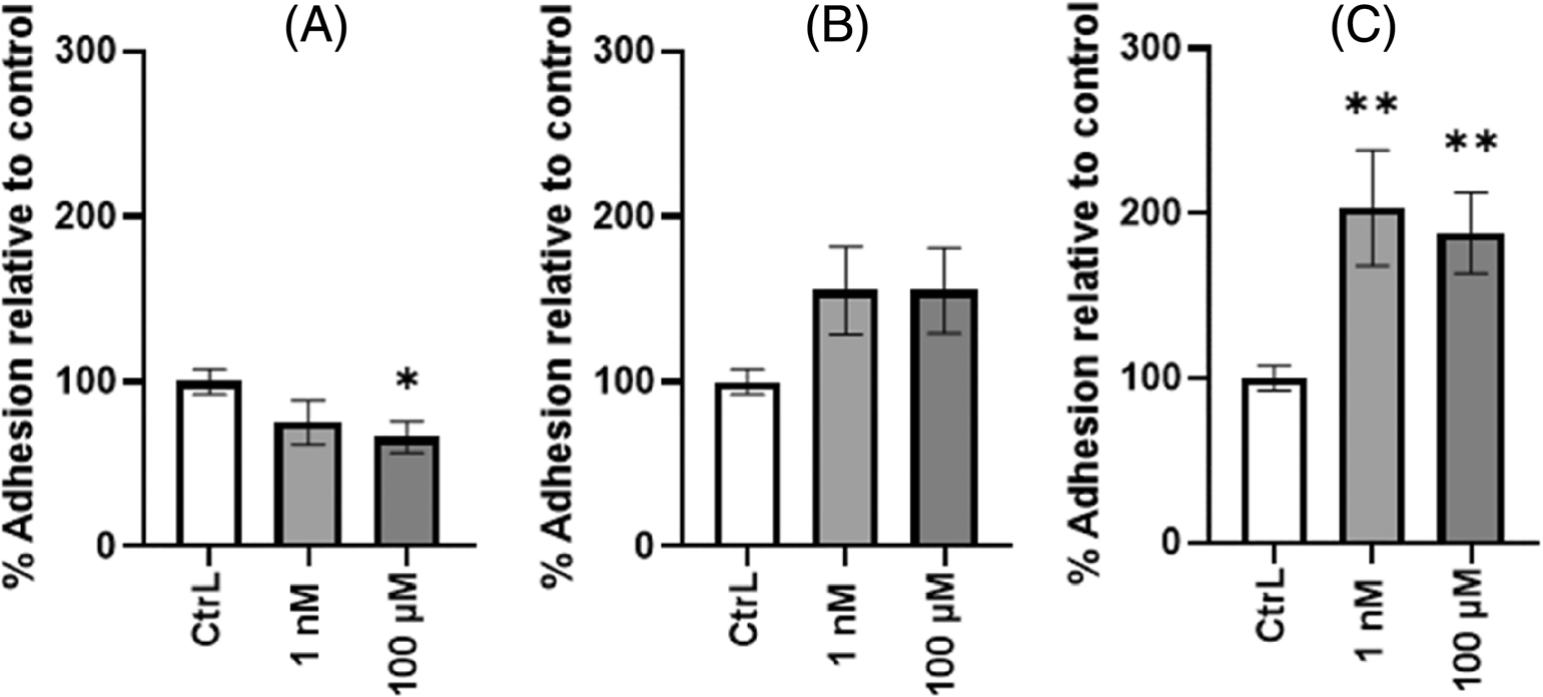
Effect of continuous exposure to endocrine disruptors on the adhesion of *P. aeruginosa* to A549 cells. Percentage of attached bacteria after 3 h of infection in the presence of 1 nM or 100 μM of BPA (A), DBP (B) and EP (C) were assessed by CFU counts. Adhesion tests were performed at MOI of 10. Results are normalised to the (CtrL) ± SEM and expressed as percent adhesion (%). Statistical analysis was performed using the non‐parametric Kruskal–Wallis test, * *p <* 0.05; ** *p <* 0.01.

**FIGURE 7:**
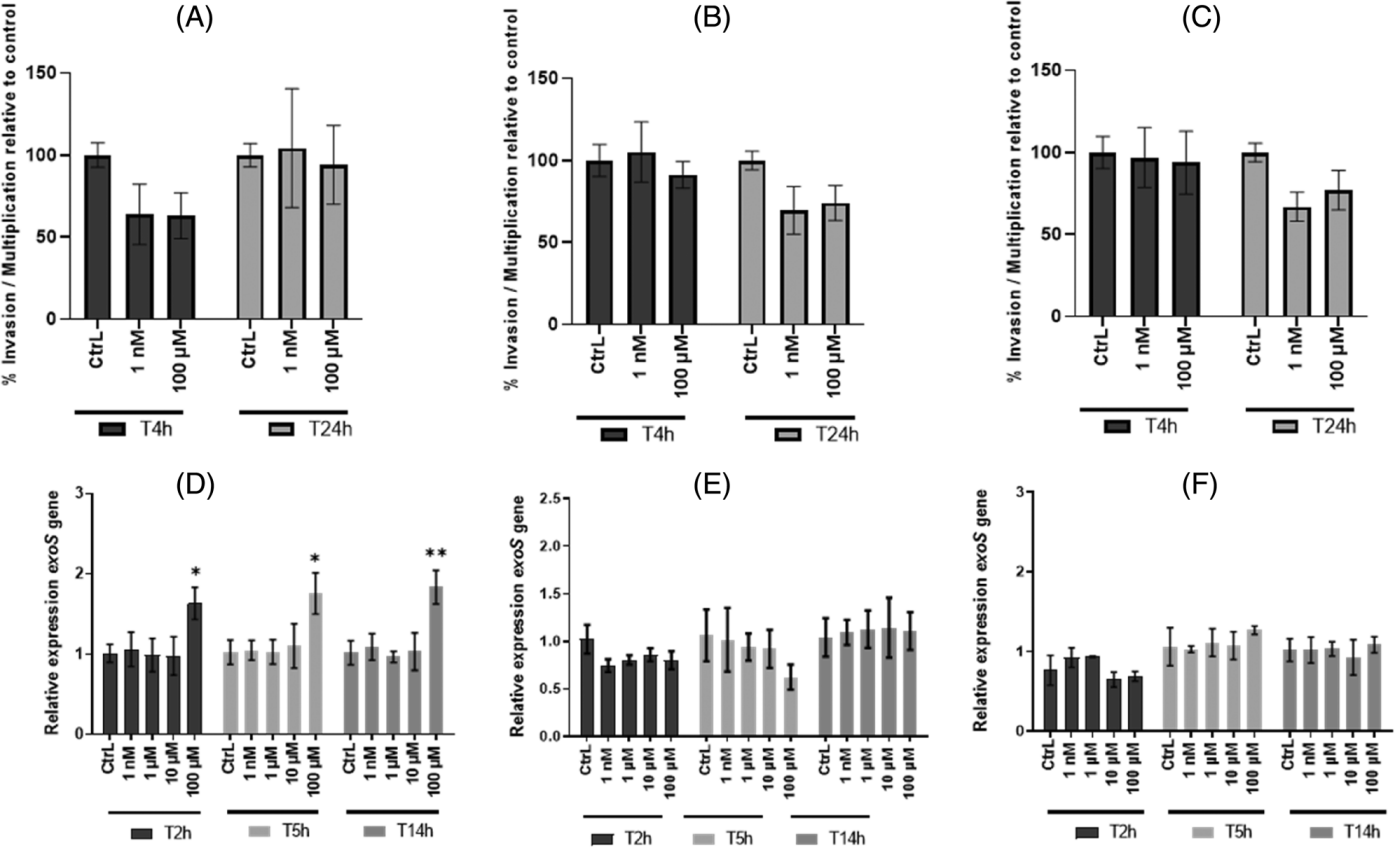
Effect of continuous exposure to endocrine disruptors on the invasion and multiplication of *P. aeruginosa* in A549 cells. (A)–(C) *P. aeruginosa* were pre‐exposed with ED (BPA, DBP EP) at concentrations 1 nM and 100 μM overnight before invasion and intracellular multiplication. The invasion (T4h) and intracellular multiplication (T24h) tests were performed on A549 cells infected at MOI10 with *P. aeruginosa* exposed to BPA (A), DBP (B) and EP (C) in DMEM supplemented with 10% SVF and with ED. The invasion test corresponds to a 3 h infection followed by 1 h treatment with gentamycin to kill extracellular bacteria (T4h). The multiplication test corresponds to a 3 h infection with a treatment maintained with gentamycin for the remaining 21 h (T24h). Invasion and intracellular multiplication results were measured by CFU intracellular counts. Results are normalised to the (CtrL) ± SEM and expressed as percent invasion or multiplication (%) Statistical analysis was performed using non‐parametric Kruskal–Wallis test, * *p <* 0.05; ** *p* < 0.01; *** *p* < 0.001; **** *p <* 0.0001. (D), (E) Relative quantification of virulence gene *exoS* expression in *P. aeruginosa* after exposure to (D) BPA, (E) DBP, or (F) EP. *P. aeruginosa* was cultured in LB medium at 37°C under agitation in presence or not of 1 nM and 100 μM of ED. Bacterial pellets were harvested at *T* = 2 h, *T* = 5 h, and *T* = 14 h mimicking the growth phases. Results are presented as mean ± SEM of triplicate. Statistical analysis was performed using 2Way ANOVA * *p <* 0.05; ** *p* < 0.01; ***p < 0.001; **** *p* < 0.0001.

Adhesion to lung epithelial cells is a crucial step in the infection process of *P. aeruginosa*. Consequently, we evaluated the effect of EDs on the adhesion capacity of *P. aeruginosa* on A549 lung cells. For these experiments, we selected only BPA, DBP and EP at 1 nM and 100 μM as they had shown significant effects on biofilm formation by *P. aeruginosa*. After 3 h post‐infection, the adhesion results were normalised to the control mean and expressed as a percentage of adhesion (Figure 6). Exposure to BPA showed a significant decrease of adhesion at 100 μM (Figure 6A). DBP showed no effect on the adhesion capacity of the bacteria to A549 cells at any tested concentration (Figure 6B). However, bacterial adhesion was significantly increased when exposed to EP at 1 nM (2.03‐fold) and 100 μM (1.88‐fold) (Figure 6C). We further investigated the impact of these EDs on the invasion and multiplication ability of *P. aeruginosa* in A549 cells. None of the ED exposures appeared to have an effect on invasion and multiplication of *P. aeruginosa* in A549 cells (Figure 7). In parallel, evaluation of the *exoS* gene expression, which encodes exotoxin S involved in apoptosis of host cells, was performed in planktonic culture. The presence of BPA (Figure 7D) at 100 μM induced a significant increase in the expression of *exoS* at different harvesting times (T2h, T5h, and T14h), but we did not observe any effect on cell viability (data not shown). No significant change in *exoS* gene expression was observed in the presence of DBP (Figure 7E) and EP (Figure 7F).

## DISCUSSION

Anthropogenic activity contributes to the release and spread of chemicals, such as EDs, in freshwater ecosystems. Moreover, the ubiquitous presence of *P. aeruginosa* in the aquatic environment is closely linked to human activity (Crone et al., 2020). That is why the interactions between the EDs found in waters and these human pathogens are to be considered. Therefore, through this study, we wish to provide new knowledge on the risks and consequences associated with ED exposure on physiology, behaviour and virulence of the pathogen *P. aeruginosa*. In addition, inter‐kingdom signalling demonstrates that human hormones can modulate and influence the phenotypes of *P. aeruginosa* by promoting virulence gene expression (Cambronel et al., 2019; Lyte, 2014; Yong et al., 2018). Since EDs are analogous to human hormones, the present work aims to demonstrate the effect of EDs—BPA, DBP, EP, MP, and TCS—on human pathogen virulence and, more specifically, on the potential modulations of the expression of virulence factors such as motility ability, siderophore production, biofilm formation, adhesion and invasion in lung cells in *P. aeruginosa*. These EDs are common representatives of four prominent ED families (bisphenols, phthalates, parabens, triclosan) commonly found in the aquatic environments to which humans are exposed in water, food and the use of personal care products.

In this study, we have reported concentrations in the nanomolar range of the five EDs studied in French surface waters (e.g., in the Nouvelle‐Aquitaine region). These observations confirm the presence of EDs in aquatic resources and consequently the risk of having bacteria exposed to EDs. However, depending on the sampling campaigns/sites, some measurements focused mainly on certain EDs, suggesting bias in the detection frequency of these molecules. In addition, the detection thresholds of the automated systems may be variable. In accordance with the ED levels encountered in French waters, in further experiments, we used the working concentrations of 1 nM, 1, 10, and 100 μM to assess the effect of EDs on the virulence of *P. aeruginosa*. These concentrations aim to mimic those found in the aquatic environment and those to which humans are exposed through water, food and personal care products (Gonsioroski et al., 2020).

The effect of exposure to EDs on the growth of *P. aeruginosa* for 24 h was first studied. It was found that EDs had no effect on the growth of the bacteria, regardless of the concentration tested. However, a delayed doubling time was observed in the presence of TCS at 100 μM. This slight variation could be explained by the biocidal effect of triclosan on the bacteria, which is known to interfere with the fatty acid biosynthetic pathway (Bibens et al., 2023) However, experiments conducted to determine minimum inhibitory concentration of EDs have shown that *P. aeruginosa* were able to grow in the presence of TCS at a concentration higher than 1 mM (data not shown).

Because of the key role of motility on the virulence of the pathogen, the effect of EDs on the swimming and swarming phenotype of the bacteria was next investigated. Swimming and swarming motility were evaluated in the presence of EDs at 1 nM, 1, 10, and 100 μM. Overall, swimming motility was decreased with EDs. In addition, TCS at 100 μM was shown to modulate the swimming phenotype of *P. aeruginosa* because a dendritic‐shaped growth area was observed. The hypothesis of a transitive switch between two swimming and swarming modes is considered, which would explain this shape, usually observed in swarm‐type motility (Kollaran et al., 2019). We think that could be a way for the bacterium to escape TCS activity. It was also found that swarm motility in the presence of MP increased. It has been demonstrated that production of extracellular tensioactive rhamnolipid molecules could affect swarming behaviour (Tremblay et al., 2007; Tremblay & Déziel, 2010). We consequently hypothesised that the presence of ED may act on surfactant production by modulating swarming movement. In addition, we demonstrated that DBP at 100 μM modulates the swarming phenotype of *P. aeruginosa* because a circular‐shaped growth area is observed. In summary, we have shown that EDs are able to modulate swimming and swarming motilities. The changes observed depend on the EDs tested and their concentrations. Except in the presence of MP, our results suggest a decrease in this virulence factor insofar as both swim and swarm were reduced.

The effect of EDs on the ability of *P. aeruginosa* to produce siderophores was next investigated. The blue‐phenazine pigment pyocyanin acts as a potent virulence factor by producing reactive oxygen species (Managò et al., 2015) that cause tissue damage and inflammation in host (Chai et al., 2013). Exposure to EDs for 24 h did not affect pyocyanin production. Pyoverdine‐mediated iron (Fe3+) acquisition serves as a signal molecule for biofilm development (Banin et al., 2005) and plays an important role in pathogenesis. It is able to act as a signal molecule for the expression of other virulence factors (exotoxin A, endoprotease)(Kang et al., 2019) (Lamont et al., 2002). Herein we show that pyoverdine production is not modulated in the presence of ED regardless of the concentration tested. Our results suggest that exposure to ED does not affect pyocyanin/pyoverdine production. Similar results of no effect of ED exposure on siderophore production have been investigated, particularly on phthalate exposure and their substitutes (Louis et al., 2022). However, these experiments were performed in a minimum medium (M9) whereas our experiments were performed in a rich medium (LB medium). We could have expected modifications in the expression of virulence factors depending on the culture medium (Fléchard et al., 2018). Nevertheless, the decrease of this virulence factor may contribute to the increase of another virulence factor such as biofilm formation. It has been reported that xenobiotic substances could favour the production of exopolysaccharide matrix (Pel) associated with repression of swim and swarm motility (Chen et al., 2014; Lewis et al., 2019).

The effects of EDs were next investigated with regard to the ability to *P. aeruginosa* to form biofilm. *P. aeruginosa* biofilm is a virulence factor as it provides protection against the host immune response (Moser et al., 2021) and antibiotic treatments given during pulmonary infection (Ciofu & Tolker‐Nielsen, 2019; Hall & Mah, 2017). In addition, exposure of *P. aeruginosa* to EDs could promote biofilm formation in humans, as some EDs such as catheters are present in medical devices (Šimunović et al., 2022). That is why we decided to address whether EDs could modulate biofilm formation in *P. aeruginosa*. Indeed, our results indicate that biofilm formation is significantly increased in presence of 1 nM BPA, DBP and EP, a concentration that we measured in the aquatic resources of the Nouvelle‐Aquitaine region. We found that the ability of *P. aeruginosa* to form biofilm when exposed to EDs differs depending on the substance considered. However, a trend toward an increase was observed for each exposure mode assessed for BPA, DBP and EP. Recent work indicates that 24 h exposure to dimethyl phthalate, di‐n‐hexyl phthalate, and di‐2‐ethylhexyl phthalate promotes biofilm formation of *P. aeruginos*a PAO1 (ATCC 15629) at concentrations between 1 and 10 μg/L (Wang et al., 2022). Similarly, exposure of *P. aeruginosa* H103 to the phthalates and their substitutes, such as 2,2,4‐trimethyl‐1,3‐pentanediol diisobutyrate (TXIB) leads to an increase in biofilm formation at 10^−3^ M (Louis et al., 2022), a concentration 10‐fold higher than the highest concentration tested in our study (10^−4^ M). These results seem to support our findings that continuous exposure to environmental concentrations of BPA, DBP and EP could increase biofilm formation in *P. aeruginosa*.

The effects of EDs were next investigated with regard to the ability of *P. aeruginosa* to infect human cells. Because we reported increased biofilm formation in presence of BPA, DBP, and EP only, we then investigated the ability of *P. aeruginosa* to adhere, invade and multiply in the cytosol of lung epithelial cells when specifically exposed to these three EDs. Experiments performed on A549 cells infected with *P. aeruginosa* indicated that exposure to EP at 1 nM promotes cell adhesion, while no effect was observed with the other tested EDs. We suggest that the presence of EP at concentrations found in water could promote the adhesion of *P. aeruginosa* to biotic surfaces, subsequently facilitating biofilm formation. However, the presence of BPA, DBP and EP did not alter the invasion and intra‐cellular multiplication abilities of the bacteria.

Finally, we have shown that exposure to EDs could modulate the expression of genes involved in the virulence of *P. aeruginosa*. Thus, four virulence genes have been selected: *exoS*, *favB*, *fliC*, and *pelE*. During infection, *P. aeruginosa* is able to inject effectors such as ExoS (exotoxin S) into the cytoplasm of cells via the type 3 secretion system (T3SS). This exotoxin is capable of reorganising the actin cytoskeleton and inducing apoptosis in cells (Kaminski et al., 2018). In cystic fibrosis patients, the implantation of *P. aeruginosa* leads the bacteria to adopt a characteristic evolutionary lifestyle and organise it into a biofilm. Pel is one of three exopolysaccharides (along with Psl and Alginate) that play important roles in the initiation of surface attachment and ensure the development and structural maintenance of *P. aeruginosa* biofilm matrix. (Colvin et al., 2012). The *fliC* gene encodes flagellin, which is an essential virulence factor for colonisation (Kazmierczak et al., 2015). FliC protein also activates innate immune responses via its recognition by Toll‐like receptor 5 in the host (Balloy et al., 2007). Finally, the *fabV* gene provides a pleiotropic role for the bacterium by conferring natural resistance to triclosan (Zhu et al., 2010). Relative expression measurements obtained by qPCR and increased expression levels of the virulence genes were obtained in the presence of the highest concentration, 100 μM. Nevertheless, in the environment, exposure to a single pollutant/ED is rare and bacteria are likely to be exposed to mixtures of compounds. Exposure to BPA and EP contributed to the overexpression of *pelE* gene. However, the increase of biofilm formation for BPA and EP conditions was significant in the presence of environmental concentration (1 nM) of EDs. Surprisingly, an increase of *pelE* expression was observed in *P. aeruginosa* exposed to TCS at high concentration and not at environmental concentrations (1 nM). It bears mentioning that the relative expression of virulence genes was not performed on biofilm but on planktonic culture in a liquid medium under agitation. The presence of BPA leads to an increase in the expression of *exoS* gene during the growth phase. However, in vitro tests conducted on A549 cells did not show any difference when *P. aeruginosa* is exposed to BPA. Although the physiological responses observed in this work are in contradiction with the results obtained for virulence gene expression, we do not rule out the role of post‐transcriptional modifications (Gaviard et al., 2019).

Overall, the results of this work highlight not only the presence of EDs at low concentrations in aquatic resources but also the consequences of exposure to these substances on the physiology and behaviour of the pathogen *P. aeruginosa*. This study reported the effect of five EDs on several virulence factors, which play major roles in survival, colonisation, adhesion, and tolerance (biofilm) of the bacteria. We have demonstrated that exposure to EDs modulates virulence factor expression of *P. aeruginosa*. More specifically, exposure of the pathogen to low concentrations of EP, that is, 1 nM as found in environmental water of Nouvelle‐Aquitaine, could promote biofilm formation and adhesion to biotic surfaces. The results of this study confirm the effects of EDs at non‐monotonic doses with greater effects at low doses (1 nM) than those observed at high doses (100 μM) as already demonstrated in human health (Lagarde et al., 2015). We believe that expression of these virulence factors at the expense of others, such as motility, can be enhanced by the presence of certain EDs, and more specifically EP.

## CONCLUSION

Taken together, these results suggest that the EDs found in aquatic environments could modulate the behaviour of *P. aeruginosa*. Indeed, we reported that exposure to BPA, DBP, and EP induces a physiological response of the bacteria and increases their ability to form biofilm and to adhere to lung cells. EP seems to be an interesting candidate for further investigation and we suggest that it could promote adhesion at the expense of motility, facilitating implantation and colonisation of the pathogen in its host. The molecular mechanisms leading to these phenotypes will require extended experiments to be deciphered. Through this work, we wish to highlight the consequences of exposure to EDs on the behaviour of the pathogens found in water resources and the associated risks to human health. We also wish to sensitize public health actors and manufacturers to the need to limit diffusion of these substances and to adopt another mode of consumption.

## AUTHOR CONTRIBUTIONS

**Audrey Thiroux:** Conceptualization (equal); data curation (equal); formal analysis (equal); investigation (equal); methodology (lead); writing – original draft (lead); writing – review and editing (equal). **Jérôme Labanowski:** Data curation (equal); writing – review and editing (equal). **Nicolas Venisse:** Writing – review and editing (equal). **Stéphanie Crapart:** Methodology (equal). **Chloé Boisgrollier:** Methodology (supporting). **Carlos Linares:** Methodology (supporting). **Jean‐Marc Berjeaud:** Conceptualization (equal); funding acquisition (lead); investigation (equal); methodology (equal); project administration (equal); supervision (equal); validation (equal); writing – original draft (equal); writing – review and editing (equal). **Romain Villéger:** Conceptualization (equal); investigation (equal); methodology (supporting); supervision (equal); validation (equal); visualization (equal); writing – original draft (equal); writing – review and editing (equal). **Alexandre Crépin:** Conceptualization (equal); investigation (equal); methodology (supporting); supervision (equal); validation (equal); visualization (equal); writing – original draft (equal); writing – review and editing (equal).

## CONFLICT OF INTEREST STATEMENT

The authors declare that they have no competing interests.

## Supporting information


**Supplementary Table 1:** Endocrine disruptor concentrations in water in the Nouvelle‐Aquitaine region.

**Supplementary Table 2:** Forward and reverse primers for each virulence gene of *Pseudomonas aeruginosa*.

## Data Availability

The data that supports the findings of this study are available in the supplementary material of this article.
