# Supplementary material for: Exposure to endocrine disruptors promotes biofilm formation and contributes to increased virulence of Pseudomonas aeruginosa
Source: Environ Microbiol Rep. 2023 Aug 16;15(6):740–56. doi: 10.1111/1758-2229.13190 (PMC10667657; doi:10.1111/1758-2229.13190)
Supplement: Supplementary file 2 — Supplementary Table 2: Forward and reverse primers for each virulence gene of Pseudomonas aeruginosa. [file EMI4-15-740-s001.pdf]

Table S2

	Forward Primer (5'-3')	Reverse primer (5'-3)	Lengh (pb)	Function
Virulence gene of *Pseudomonas aeruginosa*				
exoS	TCTACACCGGCATTCACTA	CACGGAAAGTCTTCACTACC	147	exoenzyme S
fabV	AACCTTTACGAATTGACCGA	TTGACGTCCTGCTCGTA	98	enoyl-acyl-reductase
fliC	CAGTCCACCAATATCCTGC	GTATCGGAGATACGGGTCA	137	flagellin
pelE	*TGATCGGAGCGGTCG*	*CTGAACATCATGTCGAGGC*	142	extracellular polysaccharide (biofilm)
rpoS Reference gene	AACCTTCACCCGAAGAAATC	AAGAGAGACGTCTACCGAAG	104	sigma factor
